# DAILY ENGAGEMENT IN MEANINGFUL ACTIVITY FOR HOME CARE PATIENTS WITH SUBJECTIVE COGNITIVE DECLINE AND CAREGIVERS

**DOI:** 10.1093/geroni/igad104.3742

**Published:** 2023-12-21

**Authors:** Yvonne Lu, Jennifer Ellis, Susan Perkins, Susan Hickman, Pei-Shiun Chang, Joan Haase, Laurie Otis, Rebecca Winton

**Affiliations:** Indiana University School of Nursing, Indianapolis, Indiana, United States; Aveanna Healthcare, Atlanta, Georgia, United States; Indiana University School of Medicine, Indianapolis, Indiana, United States; Indiana University, Indianapolis, Indiana, United States; Indiana University School of Nursing, Bloomington, Indiana, United States; Indiana University School of Nursing, Indianapolis, Indiana, United States; Aveanna Healthcare, Atlanta, Georgia, United States; CenterWell Home Health, Atlanta, Georgia, United States

## Abstract

Patients with subject cognitive decline (SCD) often lose sense of control, disengage from meaningful activities, and have less confidence in their ability to manage daily challenges. The objectives of pragmatic pilot phase study were to evaluate the feasibility and benefits of a Daily Engagement in Meaningful Activity Professional (DEMA-Pro) for patients with SCD; and explore nurses’ experience of DEMA-Pro implementation. Forty-nine patients at four home healthcare sites received six weekly DEMA-Pro telephone sessions. The Outcome and Assessment Information Set-D were collected at starting date (pre-intervention) and discharge date (post-intervention). Quantitative surveys and qualitative focus group methods were used to explore the DEMA-Pro nurses (n = 3) experiences. The consent rate was 67.1%, the completed intervention rate 36.7%, and the partial completed intervention rate 25.5%. For 36 subjects with discharge data available, both IADLs and self-care scores improved (d = 3.11 and d = 2.66, respectively). Specifically, those that completed all DEMA-Pro sessions (n=14), partial completers (n=12), and non-completers (n=10), had improved scores on IADLs (d = 4.0, 4.2, and 2.5, respectively) and Self-Care (d = 3.7, 3.1 and 2.0, respectively). Completers had greater improvement than non-Completers for both outcomes and greater improvement on IADLs than partial completers (all p-values < 0.03). Nurses reported high satisfaction with their training, and high confidence that the implementation of the intervention met patient and caregiver needs. The DEMA-Pro has shown benefits and feasibility that will need further testing in a large pragmatic trial in homecare settings.

